# Presentation and Outcome of Tuberculous Meningitis in a High HIV Prevalence Setting

**DOI:** 10.1371/journal.pone.0020077

**Published:** 2011-05-19

**Authors:** Suzaan Marais, Dominique J. Pepper, Charlotte Schutz, Robert J. Wilkinson, Graeme Meintjes

**Affiliations:** 1 Institute of Infectious Diseases and Molecular Medicine, University of Cape Town, Cape Town, South Africa; 2 Infectious Diseases Unit, GF Jooste Hospital, Cape Town, South Africa; 3 Department of Medicine, University of Cape Town, Cape Town, South Africa; 4 Department of Internal Medicine, University of Mississippi Medical Center, Jackson, Mississippi, United States of America; 5 Division of Medicine, Imperial College London, London, United Kingdom; 6 Division of Mycobacterial Research, MRC National Institute for Medical Research, London, United Kingdom; The University of Melbourne, The Murdoch Childrens Research Institute, Australia

## Abstract

**Background:**

*Mycobacterium tuberculosis* is a common, devastating cause of meningitis in HIV-infected persons. Due to international rollout programs, access to antiretroviral therapy (ART) is increasing globally. Starting patients with HIV-associated tuberculous meningitis (TBM) on ART during tuberculosis (TB) treatment may increase survival in these patients. We undertook this study to describe causes of meningitis at a secondary-level hospital in a high HIV/TB co-infection setting and to determine predictors of mortality in patients with TBM.

**Methods:**

A retrospective review of cerebrospinal fluid findings and clinical records over a six-month period (March 2009–August 2009). Definite, probable and possible TBM were diagnosed according to published case definitions.

**Results:**

TBM was diagnosed in 120/211 patients (57%) with meningitis. In 106 HIV-infected patients with TBM, six-month all-cause mortality was lower in those who received antiretroviral therapy (ART) during TB treatment; hazard ratio = 0.30 (95% CI = 0.08–0.82). Factors associated with inpatient mortality in HIV-infected patients were 1) low CD4^+^ count at presentation; adjusted odds ratio (AOR) = 1.4 (95% confidence interval [CI] = 1.03–1.96) per 50 cells/µL drop in CD4^+^ count and, 2) higher British Medical Research Council TBM disease grade (2 or 3 versus 1); AOR = 4.8 (95% CI = 1.45–15.87).

**Interpretation:**

Starting ART prior to or during TB treatment may be associated with lower mortality in patients with HIV-associated TBM. Advanced HIV and worse stage of TBM disease predict in-hospital mortality in patients presenting with TBM.

## Introduction

Meningitis causes significant mortality and morbidity in HIV-infected persons [1]–[4]. Tuberculous meningitis (TBM) accounts for a substantial proportion of cases, particularly in high tuberculosis (TB) prevalence areas [3]. Globally, access to antiretroviral therapy (ART) is rapidly increasing due to ART rollout programs [5]. Starting ART during TB treatment is associated with reduced mortality in HIV/TB co-infected patients [6], [7]. However, few studies have reported the influence of ART on the outcome of patients with HIV-associated TBM [8]–[10]. In this study, we report the causes of meningitis at a secondary-level hospital in a high HIV/TB prevalence setting in the era of increasing availability of ART. We also describe the presentation and outcome of patients with TBM and investigate the predictors of mortality (including ART) in these patients.

## Methods

### Ethics statement

The ethics committee of the University of Cape Town (UCT) approved the study (REC REF 223/2010). As this was a retrospective folder review, and data were analysed anonymously outside of the clinical setting, the ethics committee of UCT waived the requirement for informed consent and informed consent was not obtained.

### Setting and population

We conducted a retrospective study at GF Jooste Hospital, a 200-bed public sector referral hospital that serves adult patients from a community of approximately 1.3 million people. This predominantly low-income, high-density population is at the epicenter of the TB/HIV pandemic; in some parts of the referral area the reported TB case notification rate exceeds 1500 cases per 100 000 people per year and the HIV seroprevalence at antenatal clinics reaches 30% [11]. All patients accessing public sector care with suspected meningitis are referred to GF Jooste Hospital for investigations, including a lumbar puncture (LP). Adult patients (≥18 years) who had a LP performed over a six-month period (1 March 2009–31 August 2009) were identified from laboratory logs and included in the study.

### Procedure

As per standard protocol at the hospital laboratory [3], cerebrospinal fluid (CSF) samples underwent macroscopic examination, protein and glucose quantification, cell count, Gram stain, and bacterial and fungal culture. India ink staining and/or Crytococcus Latex Antigen Testing (CLAT) were also performed. If the clinical presentation or initial CSF findings were suggestive of TBM (as determined by the attending clinician), Ziehl-Neelsen (ZN) staining of sediment and/or *Mycobacterium tuberculosis* (*M. tuberculosis*) culture was performed. If acid-fast bacilli (AFB) were cultured from CSF, TB polymerase chain reaction (PCR) [Genotype MTBDRplus, Hain Lifesciences]) tests were performed to further identify mycobacteria species, and to determine first-line drug susceptibility (to rifampicin and isoniazid). In cases where rifampicin-resistant organisms were identified, additional drug susceptibility testing was performed by conventional methods. Syphilis serology (venereal disease research laboratory and/or *Treponema pallidum* hemagglutination assay), cytology and viral PCR examination were performed at attending clinician's discretion.

All CSF findings were reviewed. Microbiological diagnoses (i.e. where CSF analysis identified a specific etiological cause) were documented. Clinical records of patients with ‘markedly abnormal’ CSF who did not have a microbiological diagnosis were reviewed. In line with a previous study [3], CSF was considered to be ‘markedly abnormal’ when one or more of the following were present: 1) neutrophils >5 cells×10^6^/L, 2) lymphocytes>20 cells×10^6^/L, 3) protein >1 g/L, and 4) glucose <2.2 mmol/L. Patients who did not present with symptoms and/or signs of meningitis such as headache, photophobia, seizure, vomiting, altered mental state, neck stiffness or focal neurological deficit (e.g. patients with peripheral neuropathy) and those in whom an alternative diagnosis was made (e.g. subarachnoid hemorrhage), were excluded from the analysis. Data recorded for patients with TBM included medical and treatment history prior to admission, history of the presenting complaint (s), clinical examination, results of investigations, inpatient management and admission outcome. Additional information such as date of starting ART, was obtained from primary care clinic records. We used hospital medical notes, the National Health Laboratories Service database and the electronic hospital and primary care clinic (TB and ART) attendance registers to trace patients, in order to determine outcome (alive, dead or lost to follow-up) six months after LP was performed.

Patients received standardized TB treatment according to national treatment guidelines using Directly Observed Therapy Short-course (DOTS) either at the primary care TB clinic, or delivered to home by lay health care workers [12]. The duration of TB treatment (at least six to nine months) depended on the attending clinician's discretion. Patients with a new diagnosis of tuberculosis received isoniazid, rifampin, pyrazinamide, and ethambutol for two months (dosing schedules detailed in Table S1). This was followed by rifampicin and isoniazid for at least four months. The retreatment regimen included rifampicin, isoniazid, pyrazinamide, ethambutol and intramuscular streptomycin during the initial two months of treatment, followed by rifampicin, isoniazid, pyrazinamide and ethambutol for one month, followed by rifampicin, isoniazid and ethambutol for at least five months (dosing schedules detailed in Table S2). At the time of the study, national guidelines advised ART for all patients with a CD4^+^ count of less than 200 cells/µL or World Health Organization (WHO) stage 4 disease [13]. First-line ART during this study was stavudine, lamivudine, and either nevirapine or efavirenz. Efavirenz was preferred for patients who were receiving rifampicin-based antituberculosis treatment.

### Definitions

**Definite TBM** was diagnosed when 1) AFB were seen in CSF, 2) AFB or *M. tuberculosis* was cultured from CSF or 3) *M. tuberculosis* was detected by PCR from CSF. Probable and possible TBM were diagnosed according to modified published case definitions [10], [14]. **Probable TBM** was diagnosed when: 1) a patient presented with clinical features of meningitis and 2) suggestive CSF findings of TBM (total white cell count >5 cells×10^6^/L, protein >0.45 g/L and glucose <2.2 mmol/L), plus 3) one or more of the following i) chest radiograph findings consistent with pulmonary TB, ii) an extra-meningeal specimen positive for AFB, iii) other evidence of extra-meningeal TB (e.g. abdominal ultrasound features) or iv) brain computed tomography (CT) evidence of TBM including one or more of the following: basal meningeal enhancement, hydrocephalus or infarctions. **Possible TBM** was diagnosed when: 1) a patient presented with clinical features of meningitis and either 2) four or more of the following were present i) a history of TB ii) a predominance of CSF lymphocytes (>50%), iii) illness duration of more than five days iv) CSF glucose <2.2 mmol/L, v) altered consciousness, vi) clear or yellow CSF with protein>1 g/L, vii) focal neurological signs, or 3) ‘markedly abnormal’ CSF (excluding isolated hypoglycemia) with evidence of TB elsewhere.

Patients were excluded from the probable and possible TBM groups if an alternative cause of meningitis was found, or if they improved with no treatment or alternative treatment in the absence of TB treatment. **Cryptococcal meningitis** (CM) was diagnosed when CSF India ink stain, CLAT or *Cryptococcus neoformans* culture was positive. **Bacterial meningitis** was diagnosed when: 1) bacteria were isolated from CSF or 2) a patient presented with clinical features of meningitis and i) a CSF polymorphonuclear cell predominance and showed a good response to antibacterial treatment in the absence of TB treatment, or ii) a CSF polymorphonuclear cell count >1000 cells×10^6^/L, regardless of outcome. **Viral meningitis** was diagnosed when a patient presented with clinical meningitis and: 1) a virus was identified from CSF, or 2) a CSF lymphocytic predominance and had symptom resolution in the absence of antimicrobial treatment. **Loss to follow-up** was defined as being unable to trace a patient six months after LP, using the methods described above.

### Statistical analysis

Univariate analysis was performed to 1) identify significant differences between patients who did, and did not die during hospitalization and at six months follow-up and 2) identify significant differences between patients with definite and those with probable/possible TBM. Continuous variables were compared using the Student t-test or Mann-Whitney *U* test, and categorical variables were compared by Fisher's exact test.

Variables associated with inpatient mortality (p<0.2) were evaluated using multivariate analysis. Stepwise logistic regression was used to identify variables predictive of inpatient mortality in all TBM patients (regardless of HIV status) and subsequently, in HIV-infected patients only. A Cox proportional hazard model was used to assess the association of ART started before or during TB treatment with six-month mortality in HIV-infected patients who survived hospitalization. The validity of the model's assumptions was tested with Schoenfeld residuals.

A p-value<0.05 was considered statistically significant. Time to death was summarized by use of Kaplan-Meier estimates. The statistical analyses were performed with GraphPad Prism version 5 and STATA version 10.1 software.

## Results

### Causes of ‘markedly abnormal’ CSF

**Figure 1:**
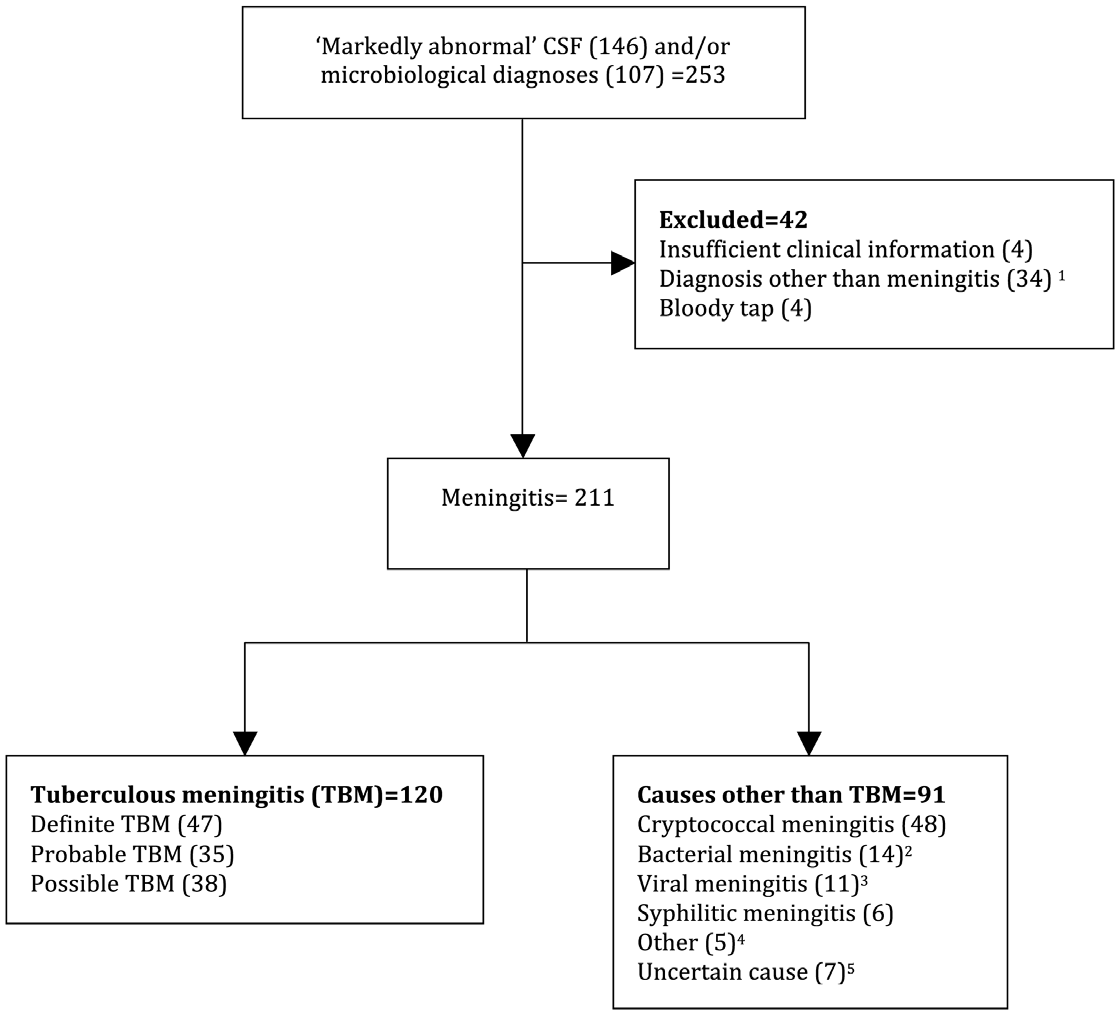
Flow diagram of differential diagnoses in patients with ‘markedly abnormal’ CSF and/or microbiological-confirmed meningitis. ^1^ Common alternative diagnoses include: hypoglycemia (n = 9), intracranial bleed (n = 7) and peripheral nerve disorders (n = 6). ^2^ Including 5 patients with CSF culture-confirmed bacterial meningitis. Organisms isolated include: *Streptococcus pneumonia* (n = 3), *beta-hemolytic Streptococcus* (n = 1), *Neisseria meningitides* (n = 1). ^3^ Including 1 patient with positive CSF polymerase chain reaction for both cytomegalovirus and herpes simplex-1 virus. ^4^ Other causes of meningitis include: Acute HIV infection (n = 1), *Toxoplasma gondii* meningoencephalitis (n = 1), disseminated Burkitt's lymphoma (n = 1), disseminated large B-cell lymphoma (n = 1), chronic resolving TBM immune reconstitution inflammatory syndrome (n = 1). ^5^ Including patients with the following differential diagnoses: 1) TBM with tuberculoma or toxoplasmosis (n = 1); 2) partially treated bacterial meningitis, viral meningitis or TBM (n = 3); and 3) viral meningitis or TBM (n = 3). CSF, cerebrospinal fluid.

During the study period, 812 LPs were performed in 698 patients. CSF analysis was ‘markedly abnormal’ (n = 146), and/or identified a cause of meningitis (n = 107) in 253 patients. Figure 1 shows the reasons for exclusion (n = 42) and diagnoses in 211 patients who were diagnosed with meningitis. The most frequent microbiological diagnoses were CM, and TBM, which accounted for 45% (48/107), and 44% (47/107), of cases respectively. Fifty-seven percent (120/211) of patients with meningitis were diagnosed with definite (n = 47), probable (n = 35) or possible (n = 38) TBM.

### Findings in patients with TBM

The demographic, clinical and investigative findings for patients with definite, probable and possible TBM are detailed in Tables 1 and 2. Eighty-eight percent of patients with TBM were HIV-infected with a median CD4^+^ count of 79 cells/uL (interquartile range [IQR] = 39–137); 20 (19%) of these patients were receiving ART at the time of presentation. The majority of TBM cases (68%) presented with advanced TBM disease (British Medical Research Council [BMRC] disease grade 2 or 3) [15] 7 days (median, IQR = 3–15 days) after symptom onset. 26/115 (23%) of patients for whom this information was available were receiving TB treatment at time of presentation for a median duration of 106 days (IQR = 50–178). Disseminated TB was common; 87/114 (76%) of patients presented with features of extra-meningeal TB. Chest radiograph abnormalities consistent with TB were observed in 74% (76/103) of patients. Abdominal ultrasound was performed in 27 patients, of which 25 (93%) showed features of TB. In addition, AFB were seen on microscopy, or *M. tuberculosis* was cultured, from one or more extra-meningeal specimens from 26 patients; specimens included sputum (n = 21), lymph node fine needle aspiration biopsy (n = 6), pleural fluid (n = 1), blood (n = 5) and urine (n = 3).

**Table 1:** Demographic and clinical characteristics of patients with definite, probable and possible tuberculous meningitis (TBM).

	Definite TBM (n = 47)		Probable TBM (n = 35)		Possible TBM (n = 38)	
Age, median (IQR)	35	(28–42)	36	(29–51)	38	(28–42)
Female, n/N (%)	22/47	(47)	16/35	(46)	22/38	(58)
HIV status, n/N (%)						
Infected	43/47	(91)	27/35	(77)	36/38	(95)
Uninfected	2/47	(4)	5/35	(14)	1/38	(3)
Unknown	2/47	(4)	3/35	(9)	1/38	(3)
CD4^+^ cell count, median (IQR)^1^	63	(35–115)	79	(36–150)	109	(33–201)
On ART at presentation, n/N (%)^2^	9/41	(22)	6/27	(22)	5/35	(14)
Previous TB, n/N (%)	15/43	(35)	7/34	(21)	12/38	(32)
On TB treatment at time of LP, n/N (%)	9/43	(21)	8/34	(24)	9/38	(24)
Symptom onset to LP, median (IQR)	7	(4–15)	6	(3–21)	3	(2–11)
Neurological symptoms, n/N (%)						
Headache	26/42	(62)	18/34	(53)	17/38	(45)
Confusion^3^	23/42	(55)	21/34	(62)	17/38	(45)
Neck pain/stiffness^3^	12/42	(29)	8/34	(24)	5/38	(13)
Nausea/vomiting	15/42	(36)	11/34	(32)	12/38	(32)
Photophobia/blurred vision/diplopia	11/42	(26)	8/34	(24)	6/38	(16)
Seizures	7/42	(17)	2/34	(6)	6/38	(16)
Neurological signs, n/N (%)						
BMRC TBM Disease Grade^4^						
1	10/42	(24)	7/34	(21)	16/38	(42)
2	29/42	(69)	23/34	(68)	20/38	(53)
3	3/42	(7)	4/34	(12)	2/38	(5)
Confusion^5^	29/42	(69)	22/34	(65)	23/38	(61)
Neck stiffness^5^	31/42	(74)	23/34	(68)	19/38	(50)*
Focal neurological signs	9/42	(21)	17/34	(50)*	2/38	(5)**

IQR, interquartile range; n, number of patients; N, total number of patients for whom results were available; HIV, human immunodeficiency virus; ART, antiretroviral therapy; TB, tuberculosis; LP, lumbar puncture; D4T, stavudine 30 mg twice daily; 3TC, lamivudine 150 mg twice daily or 300 mg daily; EFV, efavirenz 600 mg nightly; AZT, zidovudine 300 mg twice daily; NEV, nevirapine 200 mg twice daily; ddI, didanosine 400 mg daily; LPV/rtv, lopinavir/ritonavir 800/200 mg twice daily.

*Significantly different (p<0.05) from patients with definite TBM;

**p<0.01.

1 Only performed in HIV-infected patients.

2 N includes HIV-infected patients only. Treatment regimens included: 1) D4T, 3TC, EFV (n = 11), 2) AZT, 3TC, NEV (n = 3), 3) D4T, 3TC, NEV (n = 3), 4) AZT, 3TC, EFV (n = 1), 5) AZT, 3TC, LPV/rtv (n = 1), 6) AZT, ddI, LPV/rtv (n = 1).

3 Refers to symptoms reported by patient or family only.

4 British Medical Research Council TBM disease grades: 1- Glasgow coma scale (GCS) 15 with no neurological deficit; 2- GCS 11–14 without neurological deficit, or GCS 15 with focal neurological deficit; 3- GCS≤10.^15^

5 Refers to clinical findings on physical examination only.

**Table 2:** Laboratory and radiological investigation findings of patients with definite, probable and possible tuberculous meningitis (TBM).

	Definite TBM (n = 47)		Probable TBM (n = 35)		Possible TBM (n = 38)	
**Blood results**, median (IQR)						
Hemoglobin (g/dL)	10.5	(9.1–13)	12	(10.7–13.2)*	10	(8–11.4)
White cell count (cells×10^9^/L)	5.9	(4.3–8.5)	5.6	(4.5–7.7)	7.7	(5.4–10)
Sodium (mmol/L)	126	(123–130)	129	(127–133)**	130	(126–135)**
**Cerebrospinal fluid results**, median, (IQR)						
Protein (g/L)	2.6	(1.6–4.8)	2.4	(1.3–5.2)	1.2	(0.8–1.9)**
Glucose (mmol/L)	1.6	(0.9–2.4)	1.9	(1.3–2.8)	2.7	(2.1–3.2)**
Lymphocytes (cells×10^6^/L)	77	(23–199)	59	(23–143)	12	(0–31)**
Polymorphonuclear cells (cells×10^6^/L)	7	(0–39)	12	(0–12)	0	(0–3)**
**Features of TB elsewhere**, n/N (%)	35/42	(83)	21/34	(62)*	31/38	(82)
Chest radiograph abnormalities	34/38	(89)	18/27	(67)*	24/38	(63)
Abdominal ultrasound abnormalities^1^	6/6	(100)	11/11	(100)	8/10	(80)
Extra-meningeal AFB on microscopy/*M.tb* cultured	9/47	(19)	5/35	(14)	12/38	(32)
**CT brain abnormalities**,						
(excluding cerebral atrophy), n/N (%)^1^	11/16	(69)	21/23	(91)^2^	4/5	(80)^3^
Hydrocephalus	4/16	(25)	6/23	(26)	0/5	(0)
Meningeal enhancement	3/16	(19)	9/23	(39)	0/5	(0)
Infarct	5/16	(31)	12/23	(52)	1/5	(20)

IQR, interquartile range; n, number of patients; N, total number of patients for whom results were available; TB, tuberculosis; AFB, acid-fast bacilli; *M.tb*, *Mycobacterium tuberculosis*; CT, computed tomography.

*Significantly different (p<0.05) from patients with definite TBM,

**p<0.01.

1 N includes total number of patients who underwent procedure.

2 Significantly more patients with probable TBM had CT brain performed compared to patients with definite TBM, p = 0.007.

3 Significantly less patients with possible TBM had CT brain performed compared to patients with definite TBM, p = 0.04.

Atypical CSF findings in patients with definite TBM (n = 47) included a polymorphonuclear cell predominance (>50% of total leucocyte count) in six (13%), a glucose level of more than 2.2 mmol/L in 13 (28%), a protein concentration of less or equal to 0.45 g/L in three (6%) and a total leucocyte count of five or less cells×10^6^/L in two (4%). No patient with definite TBM had completely normal CSF (both biochemistry and cell count). ZN staining was requested for CSF specimens from 88 patients, including 47 (100%) definite TBM, 24 (69%) probable TBM and 25 (66%) possible TBM cases. 76/88 of these specimens (86%) were insufficient for TB microscopy. Of the 12 TBM cases who had CSF direct smear examination performed, AFB were visualized in one. Cerebrospinal fluid *M. tuberculosis* culture was requested for 106 patients including 47 [100%] definite TBM, 31 [89%] probable TBM, and 28 [74%] possible TBM cases. Significantly less patients with probable and possible TBM had *M. tuberculosis* culture performed, compared to those with definite TBM, who by definition required a positive culture (p = 0.02, and p = 0.0009, respectively). Drug susceptibility testing for first-line TB drugs (rifampicin and isoniazid) was performed on 40/47 (85%) of isolates; 35 were susceptible to rifampicin and isoniazid, three were resistant to isoniazid, one was resistant to rifampicin, and one was resistant to both rifampicin and isoniazid (multidrug-resistant [MDR] organisms). The latter two patients both died during hospitalization after starting regimen 2 TB treatment (prior to the availability of *M. tuberculosis* drug susceptibility testing results). In two additional patients MDR *M. tuberculosis* strains were cultured from extra-meningeal specimens prior to admission. One of these patients presented on a MDR TB drug regimen (i.e. ethambutol, pyrazinamide, ethionamide, ofloxacin and kanamycin) and was alive at 6-months follow-up. The *M. tuberculosis* drug resistance profile was unknown for the other patient at TBM presentation; the patient died during hospitalization after starting regimen 2 TB treatment.

**Figure 2:**
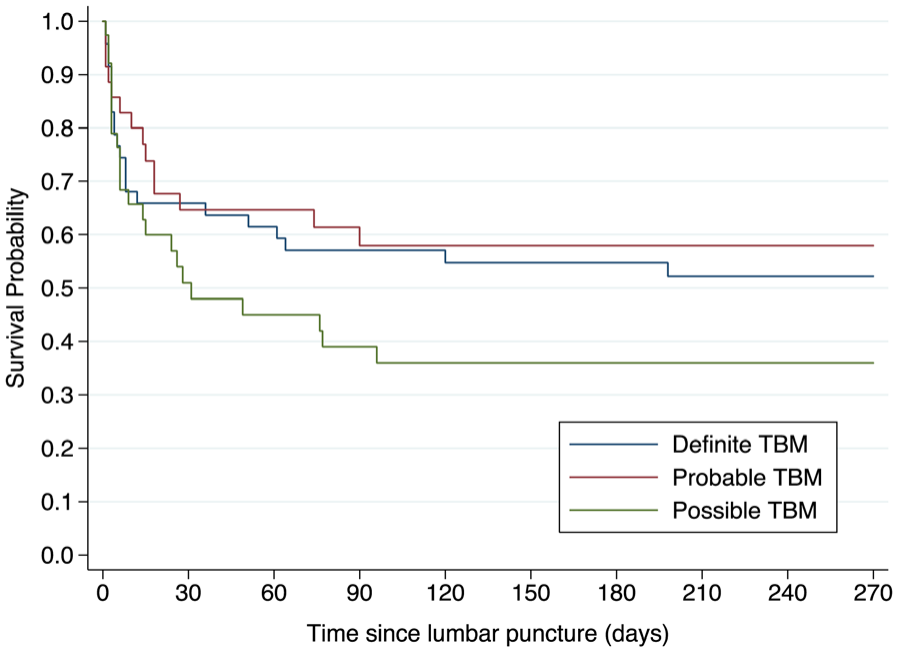
Kaplan-Meier survival curves of patients with definite, probable and possible tuberculous meningitis (TBM). Survival probability at 6-months was similar between patients with definite TBM and those with probable TBM (log-rank test p = 0.69), and possible TBM (log-rank test p = 0.15).

Table 3 describes the management and outcome in patients with TBM. Five of 89 patients not receiving TB treatment at the time of presentation, failed to initiate TB treatment after LP; all five subsequently died. In one patient with definite TBM who did not commence TB treatment, the diagnosis was not considered initially as routine CSF investigations was mildly abnormal (only abnormality: lymphocyte count = 6×10^6^/L) and there were no features of extra-pulmonary TB on chest radiograph. The remaining four patients (2-probable TBM, 2-possible TBM) died shortly after admission (within 24 hours), prior to TB drug initiation. The exact time of TB treatment initiation at presentation was known for 82/84 remaining patients: 11 patients started TB treatment 1–4 days prior to LP, but after symptom onset; 63 patients started TB treatment within 24 hours of LP; and 8 patients started TB treatment more than 24 hours after LP at a median time of three days (range, 2–8 days). Adjunctive corticosteroid treatment was started in 64/113 (57%) of patients during admission; significantly more patients with definite TBM received corticosteroids compared to those with possible TBM (71% versus 32%, p = 0.0007). The proportion of patients with probable TBM who received corticosteroids (70%) was similar to that of the definite TBM group. No patient received any other adjunctive therapy e.g. acetazolamide, or surgery. Overall inpatient mortality during hospitalization was 38% (45/120 patients), four days (median, IQR: 3–9 days) after LP (Figure 2). Among those discharged from hospital, 57% (31/54 patients) of HIV-infected patients (not on ART at time of presentation) initiated ART during six months of TB treatment. ART regimens for these patients are detailed in tables 1 and 3. At six-month follow-up, 48% of all TBM patients had died and 10% were lost to follow-up. Baseline characteristics did not differ significantly between patients who were retained in care and those who were lost to follow-up (data not shown). However, there was a trend to higher CD4+ counts in HIV-infected patients lost to follow-up compared to those who were not (median [IQR], 164 [71–250] cells/µL compared to 68 [35–144] cells/µL, p = 0.06).

**Table 3:** Management and outcome of patients with definite, probable and possible tuberculous meningitis (n = 120).

**TB treatment**		
On treatment at time of presentation, n/N (%)	26/115	(23)
Treatment started, n/N (%)^1^	84/89	(94)
Duration between symptom onset and starting treatment in days, median (IQR)	7	(3–13)
**Corticosteroids** started, n/N (%) **	64/113	(57)
**ART**		
Treatment started ≤6 months after starting TB treatment, n/N (%)^2^	31/54	(57)
Duration between diagnostic LP and starting ART in days, median (IQR)	42	(17–81)
**Outcome** 3		
Inpatient mortality, n (%)	45	(38)
Duration from LP to death in days^4^, median (IQR)	4	(3–9)
Six months, n (%)		
Alive	50	(42)
Dead	58	(48)
Lost to follow-up	12	(10)
Nine months, n (%)		
Alive	47	(39)
Dead	59	(49)
Lost to follow-up	14	(12)

TB, tuberculosis; n, number of patients; N, number of patients for whom results were available; IQR, interquartile range; LP, lumbar puncture; ART, antiretroviral therapy; TBM, tuberculous meningitis; D4T, stavudine 30 mg twice daily; 3TC, lamivudine 150 mg twice daily or 300 mg daily; EFV, efavirenz 600 mg nightly; AZT, zidovudine 300 mg twice daily; tenofovir 300 mg daily.

**Significantly more patients with definite TBM (71%) received corticosteroid treatment compared to patients with possible TBM (32%, p<0.01).

1 N includes patients not on TB treatment at presentation.

2 N includes HIV-infected patients not on ART at presentation who survived admission. Treatment regimens included: 1) D4T, 3TC, EFV (n = 14) 2) AZT, 3TC, EFV (n = 7) 3) 3TC, TDF, EFV (n = 3). Treatment regimes were not known for 7 patients.

3 Outcomes reported for all patients (n = 120) with TBM.

4 Only including patients who died during hospitalization.

Table 4 shows factors analyzed for association with inpatient mortality for all patients [n = 120] in univariate analysis. A higher BMRC TBM disease grade (2 or 3 versus 1: AOR [95% CI] = 3.0 [1.08–8.40], p = 0.04) remained predictive of mortality in multivariate analysis (logistic regression model p = 0.007, R^2^ = 0.12). Table 5 shows factors analyzed for association with inpatient mortality for HIV-infected patients [n = 106] only. CD4^+^ count (for every 50 cells/µL drop in CD4^+^ count: AOR [95% confidence interval [CI]] = 1.4 [1.03–1.96], p = 0.03) and a higher BMRC TBM disease grade (2 or 3 versus 1: AOR [95% CI] = 4.8 [1.45–15.87], p = 0.01) remained predictive of mortality in multivariate analysis (logistic regression model p = 0.01, R^2^ = 0.14).

**Table 4:** Univariate analysis of variables associated with inpatient mortality in all patients with definite, probable and possible tuberculous meningitis (n = 120).

	Died (n = 45)		Survived (n = 75)		P-value	OR^1^	(95% CI)
Age, median years (IQR) (N = 120)	37	(28–41)	35	(28–44)	0.97	-	-
Female, n (%) (N = 120)	24	(55)	36	(48)	0.71	1.2	(0.59–2.60)
History of previous TB, n (%) (N = 115)	13	(30)	21	(29)	1.00	1.1	(0.46–2.40)
On TB treatment at time of LP, n (%) (N = 115)	9	(21)	18	(25)	0.66	0.79	(0.32–1.97)
HIV-infected, N (%) (N = 114)	39	(98)	67	(91)	0.26	4.1	(0.48–34.38)
BMRC TBM disease grade 2 or 3, n (%)(N = 114)	36	(84)	45	(63)	0.03*	3.0	(1.16–7.63)
Definite TBM, n (%) (N = 120)	18	(40)	29	(39)	1.00	1.1	(0.50–2.25)
WCC, median cells×10^9^/L (IQR)	6.2	(4.5–8.9)	6.0	(4.6–9)	0.83	-	-
Hemoglobin, median g/dL (IQR)	10.3	(8.8–12.3)	11	(9.6–12.8)	0.16	-	-
Serum sodium, median mmol/L (IQR)	127	(124–133)	129	(125–134)	0.48	-	-
CSF polymorphs, median cells×10^6^ (IQR)	0	(0–14)	0	(0–14)	0.61	-	-
CSF lymphocytes, median cells×10^6^ (IQR)	39	(8–144)	46	(16–125)	0.78	-	-
CSF protein, median g/L (IQR)	2.28	(1.51–4.87)	1.76	(1.05–3.08)	0.11	-	-
CSF glucose, median mmol/L (IQR)	1.8	(1–2.8)	2.2	(1.5–2.9)	0.19	-	-
Symptoms to TB treatment, median days (IQR)	7	(2–12)	6	(4–14)	0.37	-	-
Corticosteroids started, n (%)(N = 113)	21	(50)	43	(61)	0.33	0.7	(0.30–1.41)

n, number of patients; N, total number of patients for whom analysis was performed; IQR, interquartile range; TB, tuberculosis; LP, lumbar puncture; BMRC, British Medical Research Council; WCC, total blood white cell count ; CSF, cerebrospinal fluid.

p-value statistically significant (<0.05).

1 Odds ratios (OR) and 95% confidence intervals (95%CI) reported for categorical variables.

**Table 5:** Univariate analysis of variables associated with inpatient mortality in HIV-infected patients with definite, probable and possible tuberculous meningitis (n = 106).

	Died (n = 39)		Survived (n = 67)		P-value	OR^1^	(95% CI)
Age, median years (IQR) (N = 106)	37	(28–41)	34	(28–44)	0.88	-	-
Female, n (%) (N = 106)	19	(49)	38	(57)	0.54	0.7	(0.33–1.60)
History of previous TB, n (%) (N = 102)	13	(35)	19	(29)	0.66	1.3	(0.55–3.10)
On TB treatment at time of LP, n (%)(N = 101)	9	(24)	17	(27)	1.00	0.88	(0.35–2.26)
On ART at time of LP, n (% of HIV-infected) (N = 103)	8	(22)	12	(18)	0.80	1.2	(0.46–3.38)
CD4 at presentation, median cells/µL (IQR)	54	(23–143)	109	(40–170)	0.03*	-	-
BMRC TBM disease grade 2 or 3, n (%) (N = 100)	30	(81)	39	(62)	0.07	2.64	(1.002–6.94)
Definite TBM, n (%) (N = 106)	16	(41)	27	(40)	1.00	1.03	(0.46–2.30)
WCC, median cells×10^9^/L (IQR)	6.2	(4.4–8.9)	5.6	(4.4–8.9)	0.60	-	-
Hemoglobin, median g/dL (IQR)	9.9	(8.5–11.5)	10.9	(9.4–12.6)	0.09	-	-
Serum sodium, median mmol/L (IQR)	127	(124–134)	129	(125–134)	0.96	-	-
CSF polymorphs, median cells×10^6^ (IQR)	0	(0–16)	0	(0–12)	0.92	-	-
CSF lymphocytes, median cells×10^6^ (IQR)	42	(7–135)	46	(10–130)	0.80	-	-
CSF protein, median g/L (IQR)	2.01	(1.26–3.00)	1.72	(1.04–3.09)	0.48	-	-
CSF glucose, median mmol/L (IQR)	1.93	(0.9–2.8)	2.2	(1.6–2.9)	0.22	-	-
Symptoms to TB treatment, median days (IQR)	7	(2–14)	6	(4–14)	0.40	-	-
Corticosteroids started, n (%)(N = 99)	19	(53)	36	(57)	0.68	0.84	(0.36–1.91)

n, number of patients; N, total number of patients for whom analysis was performed; IQR, interquartile range; TB, tuberculosis; LP, lumbar puncture; ART, antiretroviral therapy; BMRC, British Medical Research Council; CD4, CD4^+^ cell count; WCC, total blood white cell count ; CSF, cerebrospinal fluid.

*p-value statistically significant (<0.05).

1 Odds ratios (OR) and 95% confidence intervals (95%CI) reported for categorical variables.

**Figure 3:**
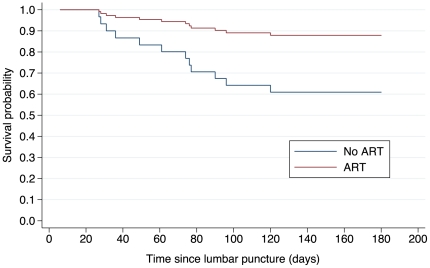
Cox proportional hazard model survival curves. **ART**: HIV-infected tuberculous meningitis (TBM) patients either on antiretroviral therapy (ART) at TBM presentation or started on ART during subsequent 6 months of antituberculosis (TB) treatment (n = 43). **No ART**: HIV-infected TBM patients not on ART at presentation nor started on ART during subsequent 6 months of TB treatment (n = 23). The model only included patients who survived hospitalization (n = 66). Hazard ratio for patients on ART = 0.30 (95% confidence interval 0.08–0.82, p-value = 0.03).

Analysis of factors associated with six-month mortality is reported only for HIV-infected hospital survivors for whom outcome was known at six-months follow-up (n = 56, Table 6). Being either on ART at presentation, or having started ART during TB treatment, was negatively associated with six-month mortality (OR = 0.2, 95% CI = 0.05–0.81, p = 0.03). This association was confirmed in a Cox proportional hazard model (n = 66), which included 10 HIV-infected patients who were lost to follow-up after discharge (Figure 3, hazard ratio = 0.30, 95% CI = 0.08–0.82, p = 0.03). No additional factors were associated with six-months mortality when all patients (regardless of HIV-status) with a known outcome at six-month follow-up were included in analysis (data not shown).

**Table 6:** Univariate analysis of variables associated with six-month mortality in HIV infected patients with definite, probable and possible tuberculous meningitis (n = 56).^1^

	Died (n = 12)		Survived (n = 44)		P-value	OR^2^	(95% CI)
Age, median years (IQR) (N = 56)	36	(29–49)	34	(28–44)	0.58	-	-
Female, n (%) (N = 56)	3	(25)	19	(43)	0.33	0.4	(0.10–1.85)
History of previous TB, n (%) (N = 55)	5	(42)	13	(30)	0.50	1.6	(0.44–6.17)
On TB treatment at time of LP, n (%) (N = 54)	4	(33)	11	(26)	0.72	1.4	(0.36–5.62)
On ART at time of LP, n (% of HIV-infected) (N = 56)	1	(8)	10	(23)	0.42	0.3	(0.35–2.70)
CD4 at presentation, median cells/µL (IQR)	98	(18–160)	104	(46–159)	0.46	-	-
BMRC TBM disease grade 2 or 3, n (%)(N = 54)	6	(50)	26	(62)	0.52	0.6	(0.17–2.24)
Definite TBM, n (%) (N = 56)	3	(25)	20	(45)	0.32	0.4	(0.10–1.68)
WCC, median cells×10^9^/L (IQR)	6.8	(4.8–9.4)	5.6	(4.1–9.4)	0.56	-	-
Hemoglobin, median g/dL (IQR)	9.7	(8.4–12.2)	10.6	(8.9–12.2)	0.60	-	-
Serum sodium, median mmol/L (IQR)	134	(126–135)	128	(123–131)	0.09	-	-
CSF polymorphs, median cells×10^6^ (IQR)	0	(0–22)	2	(0–12)	0.42	-	-
CSF lymphocytes, median cells×10^6^ (IQR)	17	(0–67)	79	(19–172)	0.06	-	-
CSF protein, median g/L (IQR)	1.28	(0.80–2.42)	1.91	(1.08–4.09)	0.12	-	-
CSF glucose, median mmol/L (IQR)	2.2	(1.5–2.9)	2.3	(1.7–3.2)	0.97	-	-
Symptoms to TB treatment, median days (IQR)	8	(4–48)	7	(4–17)	0.74	-	-
Corticosteroids started, n (%)(N = 54)	5	(42)	25	(60)	0.33	0.5	(0.13–1.79)
ART started prior to LP/during TB treatment, n (%) (N = 56)	5	(42)	34	(77)	0.03*	0.2	(0.05–0.81)

n, number of patients; N, total number of patients for whom analysis was performed; IQR, interquartile range; TB, tuberculosis; LP, lumbar puncture; ART, antiretroviral therapy; BMRC, British Medical Research Council; CD4, CD4^+^ cell count; WCC, total blood white cell count ; CSF, cerebrospinal fluid.

*p-value statistically significant (<0.05).

1 Analysis performed for HIV-infected patients who survived hospitalization for whom outcome was known at 6-month follow-up. One HIV-infected hospital survivor for whom ART treatment at TBM presentation was unknown excluded from analysis.

2 Odds ratios (OR) and 95% confidence intervals (95%CI) reported for categorical variables.

## Discussion

Several studies have reported CM as the most frequent cause of meningitis in HIV-infected patients [1]–[3], [16], [17]. In our study, TBM was the most common cause of meningitis (57%) when both microbiological-confirmed cases and cases diagnosed on clinical grounds were included. An earlier study conducted at our hospital (2006–2008) reported CM and TBM as the cause of microbiological-confirmed meningitis in 63%, and 28% of cases, respectively [3]. By comparison, we found TBM (44%) and CM (45%) to account for similar proportions of microbiological-confirmed cases. This change may reflect increasing ART access in the referral area (during 2009, 23 449 patients were commenced on ART in the Western Cape public health sector, compared to 19 527 patients during 2008 [*Catherine White, Western Cape ART Monitoring and Evaluation Programme- personal communication*]) and therefore fewer HIV-infected patients reaching the severity of immunosuppression associated with CM.

A higher BMRC TBM disease grade was predictive of death during hospitalization when all patients (regardless of HIV status) were included in the analysis. When the analysis was restricted to HIV-infected patients only, both a lower CD4^+^ and a higher BMRC TBM disease grade were associated with death. The predictive value of worse TBM disease on the mortality of both HIV-infected [18], [19] and uninfected [20], [21] patients is well documented. Two previous studies also found an association between low CD4^+^ count (less than 50 cells/µL [18] and less than 200 cells/µL [22]) and inpatient mortality in HIV-infected TBM patients. However, this finding has not always been reproduced [19], [23]. Other factors previously reported associated with reduced hospital survival in HIV-associated TBM include disease duration of more than 14 days [22] and infection with MDR-TB strains [18]. In our study, prolonged symptom duration was not associated with inpatient mortality. Due to the low prevalence of MDR-TB (MDR-TB isolates identified in 3 patients; 1 from CSF and 2 from extra-meningeal specimens), its influence on mortality could not be assessed.

In this study, six-month mortality in HIV-infected TBM patients was significantly lower in patients who received ART during TB treatment. As most studies in HIV-infected TBM patients thus far were conducted in patients not receiving ART [24], few have assessed the influence of ART on outcome. Torok *et al.* [10] reported lack of ART prior to or during TB treatment to be associated with earlier time to death by univariate, but not multivariate, analysis in adult TBM patients. However, a subsequent randomised trial conducted at the same site found no significant difference in nine-month mortality between HIV-infected TBM patients who started ART before or at two months of TB treatment (58% mortality), compared to the historical comparator group most of whom were not exposed to ART (67% mortality) [9], [10]. Croda *et al.* [8] found a history of ART prior to TBM presentation to be predictive of death at nine-months follow-up. The authors postulate that this surprising finding most likely related to non-compliance to ART.

Contrary to inpatient mortality rates which are generally similar between HIV-infected and uninfected patients with TBM [22], [23], [25], [26], six to nine-month outcome is substantially worse in HIV-infected patients [14], [27]–[29]. HIV-related illnesses (other than TBM) probably account for a substantial proportion of deaths after hospital discharge in HIV-infected TBM patients, particularly those not on ART. As previous studies in HIV-associated TB have shown a clear survival benefit in patients receiving ART [6], [7], it is intuitive to infer a causal relationship between ART and improved survival in our patient cohort. However, survival-bias might also have contributed to the association of ART and reduced mortality at six-month follow-up that we observed: those who survived were able to initiate ART.

Our study has several important limitations, which may have resulted in bias. Firstly, due to its retrospective nature, not all information was available in all cases. Specifically, a substantial proportion of chest radiographs (14%) were not available for review. This might have resulted in an underestimate of patients with probable TBM. The reasons for a substantial proportion of HIV-infected patients (43% of ART naïve patients who survived admission) failing to start ART after discharge could not be determined. Patients are not routinely followed-up at our facility after discharge from hospital; eligible patients are referred to their local ART clinics to start ART. Alternatively, patients who require prolonged admission may commence ART during admission to a step-down facility. Limited access to primary care clinic and step-down facility clinical records precluded the systematic collection of data regarding reasons for failing to start ART, drug toxicities and interactions, as well as co-morbidities after starting TB treatment and ART. Also, adherence to ART and the proportion of patients receiving directly observed TB treatment could not be assessed, and causes of death were not determined. Although similar to studies of TB patients previously conducted in our setting [30], [31], the loss to follow-up rate (10%) was not insubstantial. No factors predictive of loss to follow-up could be confirmed by analysis of baseline characteristics of these patients compared to those retained in care. However, a trend to a lower CD4^+^ count was observed in the latter group. Secondly, as study entry relied on CSF findings, patients with TBM who died prior to LP, and those who had a contraindication to LP based on brain CT, were not included in the analysis.

Thirdly, HIV itself often results in mild CSF abnormalities [32]. For this reason, we did not include patients with mildly abnormal CSF if a specific cause of meningitis was not found. However, it is well documented that a minority of patients with TBM may present with mildly abnormal, or completely normal CSF, especially in the context of HIV co-infection [8], [22], [23], [33]. This group of patients would have been excluded from our study if CSF TB microscopy and culture were negative. Furthermore, the decision to perform CSF TB microscopy and culture was not uniform, being based on the attending clinician' s discretion. Significantly less patients with possible and probable TBM compared to definite TBM had CSF *M. tuberculosis* culture performed; this could have resulted in the misclassification of some patients with definite TBM who might have had *M. tuberculosis* cultured had culture been performed.

Fourthly, CSF findings and neurological signs, most notably focal neurological deficits (21% definite TBM versus 5% possible TBM [p<0.01]), differed significantly between patients with definite and possible TBM. This might reflect the inclusion of some patients with alternative diagnoses such as viral meningitis as possible TBM or, alternatively, be indicative of more severe disease in patients with definite TBM. Patients with possible TBM were also less likely, whilst those with probable TBM were more likely, to undergo brain CT compared to patients with definite TBM (p = 0.04 and p = 0.007, respectively). This reflects the limited resources in our setting: brain imaging in the context of meningitis is usually prioritized to patients with severe disease or in whom an intracerebral space occupying lesion is suspected i.e. those with focal neurological deficits, severe depressed level of consciousness or seizures. Both the differences in CSF findings and the differences in the proportions of patients who underwent brain imaging between the definite and possible TBM groups could have resulted in bias, resulting in different corticosteroid prescription practices between the 2 groups (71% definite TBM compared to 32% possible TBM, [p = 0.0007]) and possibly differentially influencing outcomes. In our setting, corticosteroids are prescribed at the treating clinician's discretion.

### Conclusions

In our setting where most patients with TBM are HIV co-infected, advanced HIV and worse stage of TBM disease are poor prognostic factors. Starting ART prior to or during TB treatment may be associated with lower mortality in TBM patients co-infected with HIV.

## Supporting Information

Table S1 **Treatment of new tuberculous meningitis cases (Regimen 1).^1^**
(DOC)

Table S2 **Treatment of re-treatment tuberculous meningitis cases (Regimen 2).^1^**
(DOC)
