# Supplementary material for: Presentation and Outcome of Tuberculous Meningitis in a High HIV Prevalence Setting
Source: PLoS One. 2011 May 19;6(5):e20077. doi: 10.1371/journal.pone.0020077 (PMC3098272; doi:10.1371/journal.pone.0020077)
Supplement: Table S1 — Treatment of new tuberculous meningitis cases (Regimen 1).1 (DOC) [file pone.0020077.s001.doc]

**Table S1. Treatment of new tuberculous meningitis cases (Regimen 1). ^1^**

| Pre-treatment body weight | Two months initial phase | Four to seven months continuation phase ^2^ | |
| --- | --- | --- | --- |
|  | RHZE (150, 75, 400, 275) ^3^ | RH (150, 75) ^3^ | RH (300, 150) ^3^ |
| 30-37 kg | 2 tabs | 2 tabs |  |
| 38-54 kg | 3 tabs | 3 tabs |  |
| 55-70 kg | 4 tabs |  | 2 tabs |
| ≥ 71 kg | 5 tabs |  | 2 tabs |

^1^ “New” tuberculous meningitis cases: first episode of tuberculosis; regimen based on national treatment guidelines [12].

^2^ Patients were treated for a total of 6 to 9 months bases on clinician’s discretion.

^3^ Doses in milligram, taken as fixed-dose combination tablets 7 days per week.

R, rifampicin; H, isoniazid; Z, pyrazinamide; E, ethambutol; kg, kilogram; tabs, tablets
