# Supplementary material for: Presentation and Outcome of Tuberculous Meningitis in a High HIV Prevalence Setting
Source: PLoS One. 2011 May 19;6(5):e20077. doi: 10.1371/journal.pone.0020077 (PMC3098272; doi:10.1371/journal.pone.0020077)
Supplement: Table S2 — Treatment of re-treatment tuberculous meningitis cases (Regimen 2).1 (DOC) [file pone.0020077.s002.doc]

**Table S2. Treatment of re-treatment tuberculous meningitis cases. (Regimen 2). ^1^**

| Pre-treatment body weight | Two months initial phase | | 3^rd^ month initial phase | Five to six months continuation phase ^2^ | | |
| --- | --- | --- | --- | --- | --- | --- |
|  | RHZE  (150, 75, 400, 275) ^3^ | Streptomycin  (g) ^4^ | RHZE  (150, 75, 400, 275) ^3^ | RH  (150, 75) ^3^ | RH  (300, 150) ^3^ | E  400 ^5^ |
| 30-37 kg | 2 tabs | 0.5 | 2 tabs | 2 tabs |  | 2 tabs |
| 38-54 kg | 3 tabs | 0.75 | 3 tabs | 3 tabs |  | 2 tabs |
| 55-70 kg | 4 tabs | 1.0 | 4 tabs |  | 2 tabs | 3 tabs |
| ≥ 71 kg | 5 tabs | 1.0 | 5 tabs |  | 2 tabs | 3 tabs |

^1^ “Re-treatment” tuberculous meningitis cases: previously treated tuberculosis cases after cure, after completion of treatment, failure or default; regimen based on national treatment guidelines [12].

^2^ Patients were treated for a total of 8 to 9 months based on clinician’s discretion.

^3^ Doses in milligram, taken as fixed-dose combination tablets 7 days per week.

^4^ Taken 5 days per week by intra-muscular injection.

^5^ Doses in milligram, taken 7 days per week.

R, rifampicin; H, isoniazid; Z, pyrazinamide; E, ethambutol; kg, kilogram; g, gram; tabs, tablets
